# Accuracy of one algorithm used to modify a planned DVH with data from actual dose delivery

**DOI:** 10.1120/jacmp.v17i5.6344

**Published:** 2016-05-18

**Authors:** Tianjun Ma, Matthew B. Podgorsak, Lalith K. Kumaraswamy

**Affiliations:** ^1^ Department of Radiation Medicine Roswell Park Cancer Institute Buffalo; ^2^ Department of Physiology and Biophysics State University of New York at Buffalo Buffalo NY USA

**Keywords:** VMAT, 3DVH, DVH, quality assurance

## Abstract

Detection and accurate quantification of treatment delivery errors is important in radiation therapy. This study aims to evaluate the accuracy of DVH based QA in quantifying delivery errors. Eighteen previously treated VMAT plans (prostate, H&N, and brain) were randomly chosen for this study. Conventional IMRT delivery QA was done with the ArcCHECK diode detector for error‐free plans and plans with the following modifications: 1) induced monitor unit differences up to ±3.0%,2) control point deletion (3, 5, and 8 control points were deleted for each arc), and 3) gantry angle shift (2° uniform shift clockwise and counterclockwise). 2D and 3D distance‐to‐agreement (DTA) analyses were performed for all plans with SNC Patient software and 3DVH software, respectively. Subsequently, accuracy of the reconstructed DVH curves and DVH parameters in 3DVH software were analyzed for all selected cases using the plans in the Eclipse treatment planning system as standard. 3D DTA analysis for error‐induced plans generally gave high pass rates, whereas the 2D evaluation seemed to be more sensitive to detecting delivery errors. The average differences for DVH parameters between each pair of Eclipse recalculation and 3DVH prediction were within 2% for all three types of error‐induced treatment plans. This illustrates that 3DVH accurately quantifies delivery errors in terms of actual dose delivered to the patients. 2D DTA analysis should be routinely used for clinical evaluation. Any concerns or dose discrepancies should be further analyzed through DVH‐based QA for clinically relevant results and confirmation of a conventional passing‐rate‐based QA.

PACS number(s): 87.56.Fc, 87.55.Qr, 87.55.dk, 87.55.km

## I. INTRODUCTION

The delivery of an optimum dose distribution is made possible by the complex motions of the multileaf collimators (MLC) in intensity‐modulated radiation therapy (IMRT). The sharp dose falloff at the boundary between the target and neighboring organs‐at‐risk (OAR) demands appropriate quality assurance (QA) to ensure that the planned dose is delivered accurately. The advent of volumetric‐modulated arc therapy (VMAT) employing dynamic MLCs, variable dose rate, and variable gantry speed, further escalates the need for extensive quality assurance prior to treatment delivery.

Conventional gamma analysis is a powerful tool for quantitative evaluation of dose delivery with IMRT and VMAT techniques.^1^, ^2^, ^3^ This tool compares the calculated dose distributions from the treatment planning system (TPS) to the measured dose distribution from either a combination of an ion chamber and a radiographic film or detector arrays consisting of a matrix of ion chambers/diodes. Similar to gamma index (GI) analysis, distance‐to‐agreement (DTA) analysis indicates how the measured dose to the phantom agrees with planned dose in the TPS. GI searches for dose tolerance within the distance tolerance, whereas the DTA searches for the exact dose within distance tolerance. Even though GI and DTA are good indicators of deliverability of dynamic treatment plans, larger differences in a relative small volume might be overshadowed in the overall passing rate, resulting in clinically unacceptable doses to target structures and OARs.^4^, ^5^ Thus, new approaches based on measurement‐reconstructed dose distributions are being investigated to predict clinically relevant results.^(6)^

DVH‐based quality assurance (hereafter referred to as the “Planned Dose Perturbation method” used in 3DVH (Sun Nuclear, Melbourne, FL)) has been discussed by various groups recently.^7^, ^8^, ^9^, ^10^, ^11^, ^12^ Validations of this DVH‐based QA have been performed by different researchers, proving that dose reconstructed via 3DVH is consistent with dose reconstructed through other detectors and algorithms.^6^, ^13^, ^14^ Significant errors were discovered via DVH‐based QA, where the GI/DTA methods did not find the errors, indicating potential pitfalls in using recommended GI/DTA metrics and action levels.^(15)^ Significant clinical errors were observed in DVH metrics where the GI analysis incorrectly showed high pass rates.^(10)^ Positive results were also reported by the GI analysis method when numerous types of errors, including MLC positioning errors, wrong dynamic wedge angle, cold/hot spots of varied sizes, and collimator rotation errors were introduced to test the sensitivity of DVH‐based QA.^9^, ^11^, ^16^ To overcome the limitations of traditional evaluation, a hybrid QA concept, involving a combination of DVH‐based QA and GI analysis, has been recommended.^8^, ^17^

In this study, three types of clinically relevant errors were introduced into deliverable treatment plans to test the sensitivity of 3DVH software. The ability of 3DVH software to, first, detect these errors and, second, properly to account for the error through evaluation of delivered DVH, was studied.

## II. MATERIALS AND METHODS

### A. Treatment plans and error introduction

A total of 18 clinically treated VMAT plans were randomly chosen for this study. The sample included six brain plans, six prostate plans, and six head and neck (H&N) plans. All plans were created with 6 MV photon beams utilizing the Eclipse TPS version 11.0 (Varian Medical Systems Inc., Palo Alto, CA). Analytical anisotropic algorithm (AAA) and 0.2 cm grid size were used for plan calculation.

Three types of errors, namely monitor unit (MU) difference, control point (CP) deletion, and gantry angle shift, were investigated in this study. A typical full‐arc VMAT field in Eclips has 178 CPs, and each CP contains information for MLC shape, dose fraction, gantry speed, etc. In MU difference error, specific MU changes (ranging from −3% to +3% at 1% interval) were applied to each arc of the error‐free plan. This error was designed to test the sensitivity of the 3DVH system in detecting dose deviations due to changes in MU. For CP deletion study, three, five, and eight control points were deleted from each arc of the error‐free plans so as to simulate the potential data loss resulting from transferring plans via the network.^(18)^ Finally, uniform gantry angle deviations were introduced to six of the plans to test the magnitude of dose fluctuation that could potentially be introduced from gantry angle variation. MUs were changed directly in the TPS for dose‐difference errors. For the other two types of errors, the error‐free treatment plans were exported to MATLAB (MathWorks, Inc., Natick, MA) for manipulation of the control points and gantry angles. Consequently, the error‐induced plans were imported back into the TPS for validation.

### B. Dosimetric verification

Nine treatment plans were delivered on a Varian TrueBeam equipped with HD Millennium 120 MLC, while the rest were delivered on a Varian Trilogy coupled with Millennium 120 MLC (also from Varian). Most of the H&N cases were treated on the Trilogy linac, due to the field size limitations of the TrueBeam. ArcCHECK (Sun Nuclear) was used to collect measurements with cavity plug inserted as recommended by the 3DVH manual for Eclipse. Verification plans on this phantom for each patient were calculated in the Eclipse TPS from their own error‐free treatment plans. An array calibration was performed at the beginning of this study, and absolute dose calibration was measured each day prior to measurement. Four DICOM files from the TPS: radiotherapy (RT) plan, RT structure, RT dose for patient treatment, and RT dose for verification on phantom, all from error‐free plans, were sent to 3DVH. Here, the original RT doses from Eclipse were considered as standard throughout this study. Composite ArcCHECK measurements for both error‐induced and error‐free plans were saved and imported to 3DVH. The imported measurement file and DICOM files were used to reconstruct a measurement‐guided dose reconstruction on the corresponding patient structures in the 3DVH environment.

### C. Analysis

The sensitivity investigation of 2D and 3D global DTA passing rate was confined to only two acceptance criteria (3%/3 mm and 2%/2 mm) with respect to dose‐difference errors, control point deletion errors, and gantry‐angle shift errors for VMAT plans. The tolerance levels for passing rate evaluation were set to 95% for 3%/3 mm and 90% for 2%/2 mm.

#### C.1 2D DTA analysis

Two‐dimensional global DTA were obtained from comparison between measured planar ArcCHECK dose files (measured planar dose file) and Eclipse verification dose files using SNC Patient software (Sun Nuclear). Measurement uncertainty option in the SNC Patient software was not used for the analysis.

#### C.2 3D DTA analysis

A concise introduction to the dose reconstruction process in 3DVH employing ArcCHECK, specifically the ArcCHECK planned dose perturbation (ACPDP), is presented here. A detailed version is presented in the article by Nelms et al.^(19)^ ArcCHECK measurement is synchronized to each control point via recorded gantry angle information collected by virtual inclinometer at 50 ms intervals. A set of time‐resolved subbeams are established based on the ArcCHECK measurement according to their time intervals. Utilizing a predefined planned dose perturbation (PDP) model configuration (which contains a set of specific parameters to a single permutation of linac model, MLC model, and beam energy), the 3D relative dose grid is calculated for each subbeam.^(20)^ Subsequently, this 3D relative dose grid for each subbeam is morphed with the ArcCHECK cylindrical phantom via scaling factors determined by relevant entry and exit absolute doses. The summation of all the absolute subbeam dose grids, scaled by a global correction factor, gives the output of cumulative measurement‐guided dose grid on the phantom. Finally, a correction matrix is calculated as the ratios of reconstructed phantom dose and the planned phantom dose for every voxel. The voxel correction matrix is applied to the planned patient dose, resulting in the perturbed 3D patient dose. Further analysis is then carried out comparing this perturbed dose to the original patient dose from Eclipse. For 3D DTA analysis, all the data were normalized to maximum dose. A low‐dose threshold of 10% was applied; thus, dose values below 10% of the maximum value were excluded from the analysis.

#### C.3 DVH‐based analysis

Since the passing rate would not yield any clinically relevant data, reconstructed DVH curves and DVH parameters in 3DVH were generated by applying the dose distribution to patient structures and compared to corresponding parameters calculated directly from the error‐free Eclipse treatment plans. The following parameters (based on treatment anatomy) were evaluated for the DVH based study: $D_{\text{mean}}$ and $D_{95}$ for PTV; $D_{50}$ for bladder, rectum, left parotid, and right parotid; $D_{\text{max}}$ for spinal cord and optic chiasm. Percentage differences for all these DVH parameters between 3DVH and Eclipse were determined as (3DVH‐Eclipse)/Eclipse*100%.

## III. RESULTS

### A. Evaluation of dose differences: DTA versus dose‐volume parameter

All 18 error‐free plans had a composite passing rate of more than 95% for both 2D and 3D DTA evaluation using the 3%/3 mm criteria. For the 2D analysis using the 2%/2 mm criterion, four cases yielded passing rates less than 90%, whereas all cases passed the 90% passing rate criteria for 3D DTA analysis using the 2%/2 mm criteria.

The detailed results of DTA analysis for error‐induced and error‐free plans are presented in Table 1, while the number of plans passing the preset criteria (error detection rate) is shown in Table 2. For 2D 3%/3 mm analysis, over half of the −3% and +3% MU modified plans yielded passing rates less than 95%, due to fact that 3% is at the tolerance boundary. Also, the 2%/2 mm analysis detected most of the −3%/+3% MU changes. The 3D global passing rates generally gave higher values than 2D, but they decreased dramatically when the magnitude of MU errors reached +3% (two and eight cases passed 2D 2%/2 mm and 3%/3 mm, respectively, while zero and two cases passed the criteria for 3D DTA 2%/2 mm and 3%/3 mm). For −3% MU changes, the number of cases that fell into the action level was about the same.

**Table 1:** DTA passing rate summary presenting results of the DTA passing rate of all cases for dose‐difference errors, control point deletion, and gantry angle shift. In this table, averaged 2D and 3D passing rate and standard deviation for two criteria (2%/2 mm and 3%/3 mm) were presented, followed by minimum and maximum values in the bottom.

*Induced Errors*	*2D*		*3D*	
	2%/2 mm	3%/3 mm	2%/2 mm	3%/3 mm
+3% MUs	66.1±22.5	89.3±10.8 ^a^	81.6±7 ^a^	91.2±3.1 ^a^
	(30.4,97.4)	(66.7,99.9)	(68.3,89.9)	(84.9,96)
+2% MUs	77.9±17.3 ^a^	95.7±4.8	88.7±4.5 ^a^	96.2±2
	(44.7,98.9)	(86.2,100)	(79,95)	(91.9,99)
+1% MUs	87.1±11.1 ^a^	98.3±2.2	94.8±2.3	98.7±1.5
	(67.6,98.5)	(91.9,100)	(91,99.2)	(93.7,99.9)
	92.9±4.4	99.3±0.7	97.4±1.6	99.6±0.5
Error‐free	(84,98.7)	(97.6,99.9)	(93.6,99.8)	(97.8,100)
−1% MUs	92.5±4.8	98.9±1.2	96.9±1.6	99.7±0.3
	(83.3,99.7)	(95.4,100)	(94.2,99.7)	(99,100)
−2% MUs	88.6±9.1 ^a^	97.2±3.2	92.7±3.9	98.6±1.2
	(70.9,99.5)	(90.1,100)	(83.3,99.3)	(96.2,99.9)
−3% MUs	81.7±12.6 ^a^	92.9±6.8 ^a^	86.2±6.3 ^a^	95.±2.9
	(64.1,98.2)	(80.6,99.7)	(69.2,99.9)	(90.2,100)
	87.1±3.5 ^a^	94.9±2.5 ^a^	98.±0.9	99.7±0.3
3CPs deleted	(82.8,92.7)	(90.6,97.7)	(96.7,99.7)	(99.1,100)
5CPs deleted	82.4±4.5 ^a^	90.1±3.4 ^a^	96.7±1.7	99.4±0.4
	(77.2,90.1)	(85.1,93.9)	(93.3,99)	(98.6,99.9)
8CPs deleted	74.1±5.5 ^a^	84.1±4.8 ^a^	92.7±3.8	97.4±1.7
	(68.2,85.3)	(77.5,91.1)	(85.9,97.1)	(94.6,99.7)
	79.±2.9 ^a^	93.6±2.6 ^a^	97.1±1.4	99.2±0.6
+2° Gantry Shift	(75.2,82)	(89,96.5)	(95.4,99.1)	(98.3,99.9)
	79.5±4 ^a^	93.2±2.8 ^a^	96.±2.1	98.7±0.8
−2° Gantry shift	(73.3,83)	(89.3,95.2)	(93.3,99.1)	(97.5,99.9)

^a^ Indicates the number is below the corresponding criteria.

**Figure 1:**
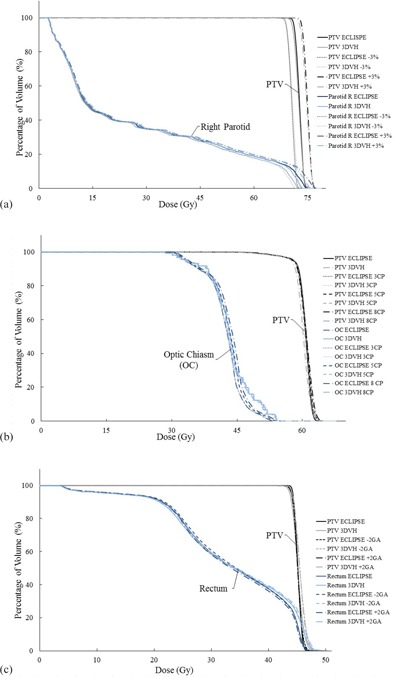
Three DVH groups (DVHs from Eclipse calculation compared to DVHs from 3DVH estimation). Each group of DVHs represent one type of error induced in this study: (a) MU difference (+3%/−3%) errors for an H&N case, (b) control point deletion errors (CP) for a brain case, and (c) gantry‐angle shift error (GA) for a prostate case. In the figures, every bold and darker line is a DVH calculated from Eclipse, whereas thinner and lighter lines are results from 3DVH analysis.

Percentage differences of selected dose‐volume parameters between 3DVH perturbation and Eclipse are listed in Table 3. Deviations in parameters between error‐induced DVH parameters and error‐free DVH parameters for PTV and chosen OARs agreed with the magnitude of the induced error in general, but slightly larger variation existed for some OARs, such as optic chiasm and spinal cord. Figure 1(a) shows an example of corresponding DVH changes for an H&N case. The bold lines (DVHs from Eclipse calculation) were aligned with corresponding thin lines (DVHs from 3DVH estimation).

**Table 2:** Rate of plans passing the preset criteria showing the ratio of number of plans passing the preset criteria (value in the bracket underneath the criteria) over the total number of plans analyzed for corresponding categories.

*Induced Errors*	*2D DTA*		*3D DTA*	
	2%/2 mm (90%)	3%/3 mm (95%)	2%/2 mm (90%)	3%/3 mm (95%)
+3% MUs	11.1%	44.4%	0.0%	11.1%
+2% MUs	33.3%	66.7%	44.4%	72.2%
+1% MUs	55.6%	94.4%	100.0%	94.4%
Error‐free	77.8%	100.0%	100%	100%
−1% MUs	72.2%	100.0%	100.0%	100.0%
−2% MUs	50.0%	72.2%	77.8%	100.0%
−3% MUs	33.3%	50.0%	22.2%	55.6%
3CPs deleted	25.0%	62.5%	100.0%	100.0%
5CPs deleted	12.5%	0.0%	100.0%	100.0%
8CPs deleted	0.0%	0.0%	75.0%	87.5%
+2° gantry shift	0.0%	33.3%	100.0%	100.0%
−2° gantry shift	0.0%	16.7%	100.0%	100.0%

**Table 3:** DVH parameter comparison summary for MU differences. Average percentage difference and standard deviation of selected dose‐volume parameters calculated by 3DVH software and Eclipse TPS for dose‐difference errors, followed by minimum and maximum values in the bracket. PTV refers to all 18 cases, while selected OARs for the corresponding six cases are: 1) rectum and bladder for prostate cases, 2) optic chiasm and brainstem for brain cases, and 3) spinal cord, left parotid, and right parotid for H cases.

*Structure*	*Dose‐volume Parameter*	*Percentage Difference*		
		−3%	*Error‐free*	+3%
PTV	$D_{\text{mean}}$	−2.7±0.9 (−4.5,0.1)	0.3±0.9 (−1.6,2.6)	3.3±1 (1.1, 5.4)
PTV	$D_{95}$	−3.1±1(−4.8,−0.3)	−0.1±0.9 (−2,2.2)	2.8±1 (0.8, 5)
Rectum	$D_{50}$	−2.6±0.7 (−3.6,−1.8)	0.4±0.8 (−0.5,1.2)	3.6±0.9 (2.2, 4.4)
Bladder	$D_{50}$	−3.1±0.9 (−4,−1.7)	0±1 (−0.9,1.5)	3.2±1.2 (1.9, 5.1)
Optic Chiasm	$D_{\text{Max}}$	−1.7±2 (−4.5,0.6)	1.3±1.9 (−1.2,3.4)	4.3±4.3 (1.6, 6.8)
Brainstem	$D_{\text{Max}}$	−0.8±2.1 (‐4.5,1.4)	2.2±2 (−1.37.5)	5.4±2 (1.9, 7.5)
Spinal Cord	$D_{\text{Max}}$	−0.5±2.4 (‐3.7,1.4)	2.4±2.1 (−0.3,4.9)	5.2±2.1 (2.3, 7.2)
Left Parotid	$D_{50}$	−2.3±2.2 (−6.1,0)	0.±2.1 (−3.7,1.8)	2.3±2.3 (−1,4.8)
Right Parotid	$D_{50}$	−2.1±2.1 (−4.6,1.1)	0.3±1.8 (−1.4,3.4)	2.6±2.3 (0.5, 6.7)

### B. DTA passing rate vs. dose‐volume parameter in detecting deleted control points

Table 1 also shows the passing rates for the control point deletion plans studied. Most of the error‐induced plans failed the 2D DTA analysis, for both 2%/2 mm criteria as well as for the 3%/3 mm criteria. As the number of deleted control points increased, the number of plans failing the criteria also increased for both 2%/2 mm and 3%/3 mm. The 2%/2 mm is more sensitive than the 3%/3 mm in detecting these errors, as seen from Table 1.

The 3D analysis did not show significant differences in pass rates when measurements of CP‐deletion‐induced plans were compared with error‐free plans from the Eclipse TPS. The average pass rates for the three‐CPs‐missing, five‐CPs‐missing, and eight‐CPs‐missing cases were above the passing criteria (90% for 2%/2 mm and 95% for 3%/3 mm, respectively), as seen from Table 1. Also the number of plans passing both the 3D DTA analysis (2%/2 mm and 3%/3 mm) is high for all CP deletion cases, as illustrated in Table 2.

The percentage difference in dose‐volume parameters between the CP‐error‐induced plans and error‐free Eclipse plans are listed in Table 4. Most of the DVH parameters did not show significant differences between the two plans even for eight CPs‐deletion plans, indicating that DVH analysis for CP deletion may not detect the errors. Differences for optic chiasm showed slight overdose compared to other structures, while the left parotid was relatively underdosed. All the other percentage differences were close to zero. Most of the 3DVH predictions agreed with the same error‐induced plan recalculated in the Eclipse TPS. An illustration of these changes can be seen in Fig. 1(b).

**Table 4:** Average percentage difference and standard deviation of selected dose‐volume parameters for control point deletion errors with minimum and maximum value in the bracket. PTV refers to four prostate cases and four brain cases, while selected OARs for corresponding cases are: 1) rectum and bladder for prostate cases, and 2) optic chiasm and brainstem for brain.

		*Percentage Difference*					
*Structure*	*DVH Parameter*	*3CPs Deleted*		*5CPs Deleted*		*8CPs Deleted*	
		*3DVH*	*Eclipse*	*3DVH*	*Eclipse*	*3DVH*	*Eclipse*
PTV	$D_{\text{mean}}$	0.4±0.5	0.2±0.3	0.1±0.6	0.2±0.5	−0.9±0.9	0.2±0.8
		(−0.4,1.2)	(−0.3,0.5)	(−1.3,1.6)	(−0.6,0.8)	(−2.3,0.4)	(−1.2,1.3)
PTV	$D_{95}$	−0.2±0.6	−0.2±0.4	−0.4±0.7	−0.7±0.9	‐1.6±1	−1.6±1.6
		(−1.1,0.9)	(−0.7,0.5)	(−1.7,1.2)	(−1.9,0.5)	(−2.9,−0.4)	(−4.1,0.5)
Rectum	$D_{50}$	0.4±0.4	0.4±0.6	−0.2±0.3	0.3±1.4	−1.1±0.5	0.4±2
		(−0.2,0.6)	(−0.5,0.7)	(−0.6,0)	(−1.6,1.4)	(−1.9,−0.6)	(−2.5,2.1)
Bladder	$D_{50}$	0.±0.9	0.5±0.4	−0.7±0.9	1.1±0.6	−0.9±1.2	2.3±1.4
		(−0.9,1)	(0, 0.8)	(−1.7,0.4)	(0.2, 1.5)	(−2.4,0.3)	(0.5, 3.8)
Optic Chiasm	$D_{\text{Max}}$	1.1±1.7	0.3±0.6	0.7±1.9	0.5±1.3	0.3±1.8	1.2±1.2
		(−0.6,0.7)	(−1,3.4)	(−2.4,2.5)	(−1.5,1.4)	(−2.1,1.8)	(−0.4,2.6)
Brainstem	$D_{\text{Max}}$	1.6±1.9	0.8±0.7	0.9±2.1	1.5±0.5	0.2±2.4	2.8±0.3
		(−1.1,3.4)	(0.1, 1.7)	(−2,3.4)	(1.1, 2.2)	(−3.1,3.6)	(2.5, 3.2)

### C. Sensitivity to gantry angle: comparison of DTA passing rates and dose‐volume parameters

The results for the 2° gantry angle shift in the clockwise (indicated by “$+2^{\circ }$”) and counterclockwise (indicated by “$-2^{\circ }$”) direction are shown in Table 1. A gantry angle shift of 2° roughly corresponds to 3 mm spatial shift on the detector level of ArcCHECK phantom. The 3D DTA analysis did not detect any errors indicated by the high passing rates for all plans, as shown in Table 2. In contrast, the 2D DTA analysis did show low pass rates for most of the cases. Table 2 also suggested that moving clockwise and counterclockwise seems to produce similar results in terms of the passing rates.

Table 5 illustrates the changes in DVH parameters due to gantry‐angle shift errors for both target and critical structures. Minor differences between error‐free plans and gantry angle shifted plans were found, mostly less than 2% (except the optic chiasm in one case). This suggests that DVH‐based analysis provides similar results as 3D DTA analysis in Table 1. Recalculation in Eclipse of the same error‐induced plans agreed with 3DVH prediction, as shown in Fig. 1(c).

**Table 5:** DVH parameter comparison summary for gantry angle shifts. Average percentage difference and standard deviation of selected dose‐volume parameters calculated by 3DVH software and Eclipse TPS for gantry‐angle shift errors with minimum and maximum value in the bracket. PTV refers to three prostate cases and three brain cases, while OARs for the corresponding cases are: 1) rectum and bladder for prostate cases, and 2) optic chiasm and brainstem for brain cases.

		*Percentage Difference*			
*Structure*	*DVH Parameter*	$+2^{\circ }$		$-2^{\circ }$	
		*3DVH*	*Eclipse*	*3DVH*	*Eclipse*
PTV	$D_{\text{mean}}$	0.9±0.6	0.1±0.1	1.1±0.7	0.±0.1
		(0.1, 1.6)	(0, 0.2)	(0.3, 2.3)	(−0.1,0.1)
PTV	$D_{95}$	0.3±0.8	−0.3±0.3	0.5±0.9	−0.3±0.3
		(−0.7,1.5)	(−0.7,0.2)	(−0.4,1.7)	(−0.7,0.2)
Rectum	$D_{50}$	1.1±0.2	−0.3±1.1	0.7±0.7	0.9±1.1
		(0.9, 1.2)	(−1.1,0.5)	(0.1, 1.2)	(0.1, 1.6)
Bladder	$D_{50}$	0.9±1	0.1±0.3	1.±0.9	0.±0.7
		(0.2, 1.6)	(−0.1,0.4)	(0.3, 1.6)	(−0.5,0.5)
Optic Chiasm	$D_{\text{Max}}$	1.8±2	−0.3±1.2	2.1±1.5	0.6±0.6
		(−0.4,3.5)	(−1.1,1.1)	(0.4, 3.4)	(−0.1,1.1)
Brainstem	$D_{\text{Max}}$	1.7±1.9	1.1±1.1	2.1±1.4	−0.5±1.4
		(−0.5,4)	(0.2, 2.4)	(0.7, 4)	(−2.1,0.6)

## IV. DISCUSSION

A 3D analysis yielded higher pass rates than 2D analysis for the error induced plans, as shown in Table 1. For example, the 2° uniform gantry angle shift represents a 3 mm shift at the level of the ArcCHECK detectors. The 2D analysis was able to detect these errors, since any spatial shift in the 2D analyzing plane will result in incorrect dose comparisons between the measured and predicted dose matrix. For 3D analysis, addition of the third dimension results in more points to search within the evaluation criteria and configuration of low‐dose threshold results in a fairly large amount of points involved in the comparison. Thus, an error within a relatively small area/volume will be neglected, resulting in higher passing rates.

As seen from Table 2, none of the eight‐CP‐deleted plans passed the 2D DTA 2%/2 mm criteria, whereas 75% of the eight‐CP‐deleted plans passed the 3D DTA 2%/2 mm criteria. Even though the beam weight of eight CPs could contribute up to 4% of the total dose per field, the detected dose might also be affected by the MLC shape, which made it hard to estimate the expected deviation from error‐free plan. The cold spot formed by the consecutive deleted CPs may be detected by the 2D DTA analysis, but might be hidden within the entire volume comparison in the 3D DTA analysis. Therefore, as shown in Table 2, 2D DTA analysis demonstrated a superior error‐detectability for all three error types studied.

Minor differences between reconstructed DVH parameters from 3DVH and TPS DVH parameters were observed as seen in 3, 4, 5. These results indicate that 3DVH can accurately predict errors in treatment delivery. However, there are small differences in the slopes of the 3DVH curves and the TPS DVH curves, as evident from Fig. 1(c). These slope differences can be a consequence of uniform built‐in machine configuration in the model library

(not our linac model) in the 3DVH, such as machine type, photon energy, and MLC model. Also, alignment of ArcCHECK before measurements might have a slight effect on the slope. Moreover, the 3DVH algorithms might also make some contributions to the DVH differences. Larger discrepancies were seen in some OARs with small volume, or to volume where the doses were reconstructed using the measurements from the end of the ArcCHECK detectors. In brain cases, as presented in Fig. 1(b), the perturbed DVH curves of optic chiasm, which had an extremely small volume, were relatively coarse due to the limited number of voxels within the structure.

Unlike the 2D analysis, the 3DVH and Eclipse recalculation did not show significant differences in the DVH parameters when comparing the error‐induced plans to the error free plans for both the CP deletion and gantry‐angle shift plans. This is evident from 4, 5. These results imply that some of the errors do not significantly affect the dose distribution within the patient. But a good QA system must detect errors in treatment planning and delivery regardless of how significant the resulting error in delivery impacts delivered dose to the patient.

## V. CONCLUSION

Detection of delivery errors is crucial in radiation therapy. The 2D DTA analysis is sensitive to detecting errors in plan delivery compared to 3D DTA analysis. DVHs from 3DVH were found to correspond to DVHs of error‐induced plans in Eclipse. Thus, any dose discrepancy or uncertainty should be further analyzed through DVH‐based QA to evaluate clinically relevant results.

## ACKNOWLEDGMENTS

The authors would like to thank the anonymous reviewers for their valuable comments.

## COPYRIGHT

This work is licensed under a Creative Commons Attribution 3.0 Unported License.
